# Usage, Pleasure, Price, and Feeling: A Study on Shopping Orientation and Consumer Outcome

**DOI:** 10.3389/fpsyg.2022.823890

**Published:** 2022-02-10

**Authors:** Shaoqiong Zhao, Pu Chen, Yan Zhu, Feng Wei, Fangmei Liu

**Affiliations:** ^1^School of Economics and Management, China University of Petroleum-Beijing, Beijing, China; ^2^School of Economics and Management, East China Jiaotong University, Nanchang, China; ^3^School of Economics and Management, Tsinghua University, Beijing, China; ^4^Institute of Internet Industry, Tsinghua University, Beijing, China; ^5^College of Management, Shenzhen University, Shenzhen, China

**Keywords:** RFT, mood, price, hedonic and utilitarian, consumer behavior

## Abstract

Understanding the behavior of consumers and especially the purchase-related behavior has been a focus of research for the past decades. Thus, researchers and practitioners are curious to know how purchase patterns are different under different conditions such as product category, price, feeling, and so on. The primary focus of this study was to examine how the price of the products influences the purchase behavior of consumers across hedonic and utilitarian categories under regulatory focus theory (RFT). The secondary insight was to examine how mood can moderate this impact. We conducted three experimental studies to examine these research questions regarding the preference of consumers of hedonic (utilitarian) products when the price is low (high) and at different mood conditions in this purchase process. The results confirmed our hypothesis that product category has a significant impact on purchase choice of products and mood can mediate this impact. In the last section, we discussed the theoretical contribution, strategic insights for product designers and marketers, and possible future research directions.

## Introduction

Academia researchers and practical business managers are always interested in what can affect consumer purchase choices. Vieira et al. (2018) studied the relationship between hedonic and utilitarian values on shopping response through meta-analysis using 190 previous studies. This shows the importance of hedonic and utilitarian nature on consumer purchase behavior, which is the motivation for conducting this study. Hedonic and utilitarian shopping values have been a hot research topic since the mid-1980s in the marketing field (e.g., Li and Katsumata, 2020; Li et al., 2021a). Dhar and Wetenbroch (2000) proposed that consumer choices are driven by utilitarian and hedonic considerations. For example, when a couple is choosing a new automobile among a bunch of automobiles, they may care about the hedonic features such as leather seats and sunroof; they may also care about the utilitarian features like miles per gallon (MPG) and safety. Hedonic refers to the fun, experiential, and aesthetic natures, and utilitarian refers to the practical, functional, and instrumental natures (Dhar and Wetenbroch, 2000; Anderson et al., 2014; Vieira et al., 2018). Products differ in the extent to which their overall attitudes are derived from hedonic and utilitarian domains. Consumers also form their attitudes with distinct hedonic and utilitarian components. Consumers then distinguish products between hedonic and utilitarian nature from these different perspectives and make purchase decisions accordingly (Batra and Olli, 1990; Baltas et al., 2017; Kivetz and Zheng, 2017; Wang et al., 2020).

In this study, we examined consumer purchase preference between two types of products: one is mainly in hedonic nature and the other is mainly in utilitarian dimension. We compared preferences for these products under two price conditions: laptops (high-price conditions) and cell phones (low-price conditions). Based on the regulatory goals and loss aversion theory literature (Luqman et al., 2021), we proposed the first hypothesis that under high-price conditions, the consumer tends to fulfill the prevention goals since loss looms bigger than gain and, under low-price conditions, the consumer tends to fulfill the promotion goals for fun. We also showed that the predicted asymmetry can be attenuated by manipulating the promotion/prevention scenarios and different mood situations.

The remainder of the study is organized as follows. In the section “Theory and Hypotheses,” we defined the terms “hedonic” and “utilitarian” and then briefly reviewed the prior research relevant to regulatory focus, loss aversion, and mood effect in decision-making. Hypotheses are formed based on the review of relevant literature. In the section “Materials and Methods,” we conducted three experimental studies to confirm the hypothesis that we proposed. Then we concluded this study with a discussion of the theoretical contribution, managerial implications, and future research directions.

## Theory and Hypotheses

### Hedonic and Utilitarian Concepts

Hedonic and utilitarian aspects of products play an important role in consumer choice (Dhar and Wetenbroch, 2000; Sun et al., 2017; Vieira et al., 2018). Hedonic products are viewed mostly as experiential consumption with pleasure orientation for fun, such as sports cars and designer clothes, whereas utilitarian products are viewed mostly as functional and instrumental consumption with practical orientation, such as microwaves and textbooks (Childers et al., 2001; Kivetz and Zheng, 2017; Wang et al., 2020).

At the attribute-specific level, we can also define each product attribute as hedonic and utilitarian (Dhar and Wetenbroch, 2000; Kivetz and Simonson, 2002; Kivetz and Zheng, 2017; Wang et al., 2020; Li et al., 2021b). Whether a product is “hedonic” or “utilitarian” is based on the relative salience of its hedonic and utilitarian attributes together. For example, the design of the bag can be viewed as a hedonic attribute, whereas the carrying stuff is a utilitarian function. Under this view, in this study, we focused on the hedonic and utilitarian aspects of products at the attribute-specific level and examined how the price of the products influence consumer choices of hedonic and utilitarian preference. These different considerations of hedonic and utilitarian attributes can influence attitudes of consumers, thus impacting the evaluations (Batra and Olli, 1990; Baltas et al.,. 2017; Kivetz and Zheng, 2017; Wang et al., 2020). Relative preference of consumers of these two benefit dimensions, namely, hedonic and utilitarian, has been a hot topic in the marketing field (Dhar and Wetenbroch, 2000; Voss et al., 2003; Okada, 2005; Baltas et al., 2017; Kivetz and Zheng, 2017; Wang et al., 2020). Consistent with this view, research presented in this study focused on hedonic and utilitarian aspects of the product on the attribute-specific level and, in this context, we examined the impact of goal orientation (prevention or promotion) on consumer choices of hedonic and utilitarian attributes.

Among these studies, Kivetz and Simonson (2002) confirmed that consumers usually assign a higher weight to the utilitarian dimension; when they believe that they have earned the right to indulge, then they make a different choice. Okada (2005) also proposed that people are more likely to have fun if the situation allows them to justify; two choice patterns are observed in typical purchase contexts driven by the need of consumers for justifying the purchase. There is converging evidence that promotion focus offers a better fit with hedonic attributes, whereas prevention focus is likely to be more compatible with the more practical and conservative utilitarian attributes (Chernev, 2004).

### Regulatory Focus Theory

Consumers usually purchase products based on whether the performance of products can meet their goals. People tend to approach pleasure and try to avoid pain (Higgins, 2012). The underlying nature of approach-avoidance motivation is defined as regulatory focus; this distinguishes self-regulation with a promotion focus or with a prevention focus. The promotion focus is concerned with aspiration, advancement, growth, and accomplishment, whereas the prevention focus is concerned with security, responsibility, and safety (Higgins, 2012). Fundamentally, different needs generate different motivations: promotion needs and prevention needs, as defined. Then the goal orientation of consumers (promotion or prevention) can be linked with the hedonic and utilitarian nature of the product attributes. There is converging evidence that “promotion focus offers a better fit with hedonic attributes, whereas prevention focus is likely to be more compatible with the more practical and conservative utilitarian attributes” (Chernev, 2004; Werth and Foerster, 2007; Li et al., 2018; Luqman et al., 2021). When consumers are with a promotion focus, they are actively looking to fulfill the promotion goals such as pleasure by the consumption of hedonic goods. While consumers are with a prevention focus, they are actively looking to fulfill prevention goals such as security and safety, which can be met by the consumption of practical and functional utilitarian products.

Fulfillment of the promotion goals by product consumption leads to the pleasure experience. Promotion-focused consumers are more likely to obtain achieving pleasure; thus, they pay more attention to hedonic attributes that can generate pleasure. Fulfillment of the prevention goals by product consumption tries to eliminate the painful experience. Prevention-focused consumers focus more on avoiding undesired outcomes; thus, they pay more attention to utilitarian attributes (Chernev, 2004; Scholer and Higgins, 2012; Cheng et al., 2013; Li et al., 2018; Luqman et al., 2021). For example, when purchasing automobiles, the promotion goals can be met by the contemporary design of the car, while the prevention goals can be met by the safety features of the car. These differences in the focus of individuals on either hedonic or utilitarian attributes lead to different importance in these attributes when evaluating the products. The promotion-oriented consumers are more likely to put more weight on hedonic (relative to utilitarian) attributes and *vice versa* for the prevention-focused consumers. Specifically, consumers expect to fulfill the promotion goals on the hedonic attributes, whereas they expect to fulfill the prevention goals on the utilitarian attributes.

### Loss Aversion

When consumers are making choices, there is always a risk accompanied by the choice. People tend to underestimate outcomes that are merely probable compared with the outcomes that will happen for sure (Kahneman and Tversky, 1979). In addition, losses loom larger than gains (Tversky and Kahneman, 1991; Novemsky and Kahneman, 2005; Gal and Rucker, 2018). Thus, people are more afraid of loss than being happy for gaining. “Hedonic consumption situations, people make decisions based on multisensory fantasies and emotional gratification” (Kim, 2016). Conversely, in utilitarian situations, “people make decisions cognitively, based on instrumental reasons while thinking about the expectations of consequences” (Kim, 2016). The consumption of hedonic goods might not lead to a pleasure experience due to the perception of different consumers incurring a loss while the consumption of utilitarian goods can offer practical and functional benefits that are perceived as positive gains always.

Based on the literature review of hedonic and utilitarian concepts, regulatory focus theory (RFT), and loss aversion theory, we developed the first part of hypotheses about choices between hedonic and utilitarian products under different price conditions. Specifically, consumers expect to fulfill the promotion goals on the hedonic attributes to get pleasure during the consumption, whereas they expect the fulfillment of prevention goals on the utilitarian attributes that offer security and safety of consumption. Besides, people are more afraid of loss than being happy with gain. The price of the product largely impacts the financial risks that consumers are facing. The potential loss of hedonic product consumption is even magnified when the price is high. In this situation, prevention focus will take lead and direct to the selection of utilitarian goods for positive gains from functional benefits. The concern of potential loss is small or the loss can be bared even there is when the price is low, thus the promotion goal will take lead and direct to the selection of hedonic goods.

H1: When the price of the products is high, the concern of loss is also higher, consumers tend to avoid potential loss by meeting the prevention goals of choosing utilitarian products.
H2: When the price of the products is low, the concern of loss is also lower, people care more about the gaining of pleasure by meeting the promotion goals of choosing hedonic products.
H3: The choice preference can be attenuated by manipulating the promotion and prevention goals of consumers.

### Mood Regulation

Research has made considerable progress in understanding affect, showing that moods, feelings, and emotions are related to nearly all aspects of consumption behavior. People regulate their affective states to achieve hedonic gratification or to achieve instrumental goals. Both forms of goals pursuit are grounded in the classic approach-avoidance paradigm: from the hedonic perspective, people try to maintain positive mood states and avoid negative mood states, while from the instrumental perspective, people seek to achieve positive goals and avoid negative outcomes (Tamir, 2005). The hedonic principle suggests that people are motivated to approach pleasure and avoid pain. Fundamentally, affect regulation is driven by the principles of approach and avoidance motivation. Two distinct regulatory systems govern how people pursue goals: promotion focus and prevention focus (Arnold and Reynolds, 2009); fulfillment of the promotion goals leads to the pleasure experience, while fulfillment of the prevention goals focuses on eliminating the painful experience. Both of the self-regulation systems can be a chronic predisposition of individuals or can be situationally induced (Aaker and Lee, 2001; Semin et al., 2005; Parsad et al., 2021).

Thus, mood can affect the choice through the two different regulatory focuses. When consumers are in a negative mood, they are motivated to improve that mood (i.e., the mood management hypothesis, Zillman, 1988), and the potential big loss with expensive purchase can significantly worsen the bad mood further, thus they tend to fulfill the promotion goals to generate the pleasure feelings; when people are in a positive mood, they are motivated to preserve that mood (i.e., the mood maintenance hypothesis, Clark and Isen, 1982), the gain in pleasure with few risk of inexpensive goods can greatly improve the mood, so they tend to fulfill the prevention goals to maintain those feelings instead of breaking it. Here, we proposed our second part of hypotheses expressed as follows:

H4: When consumers are in a good mood, they tend not to destroy it and keep it with prevention focus, so they prefer to choose utilitarian products; when consumers are in bad mood, they tend to improve it with promotion focus, so they prefer to choose hedonic products.

**FIGURE 1:**
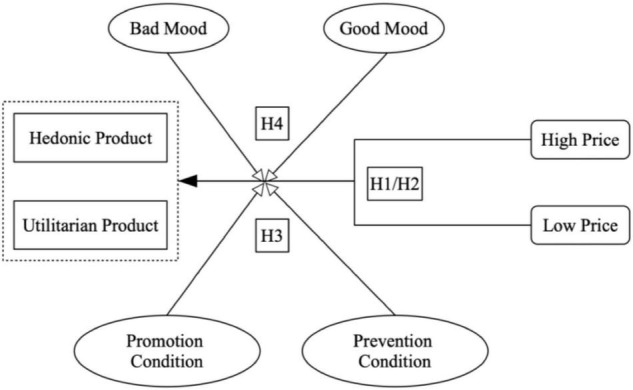
Conceptual framework.

The conceptual model of this study was developed based on the above hypotheses, which are depicted in Figure 1. Consumer preferences of hedonic or utilitarian products are different under different price conditions, and this relationship is moderated by the prevention/promotion goals and mood conditions.

## Materials and Methods

In the three experimental studies with cell phones and laptop computers, we showed that the price of the products (cost to the consumers) has an effect on consumer choice between hedonic and utilitarian products, and this effect can be attenuated by the manipulation of the goals and mood of consumers. Cell phones and laptop computers are selected since participants should be familiar with them as undergraduate students from a Midwest business school (Lubar Business School at UW-Milwaukee in the United States), and they could also imagine the products in various usage scenarios.

### Study 1: High- vs. Low-Price Choices Between Hedonic and Utilitarian Products

#### Design and Task

The purpose of this study was to testify the impact of the product price on consumer purchase choices between hedonic and utilitarian products. A 2 (product benefits: hedonic vs. utilitarian) by 2 (price: high vs. low) between-subjects design was used. A total of 120 undergraduate students participated in the study for extra credit. The between-subjects experimental design consisted of a high-price condition (laptop computers) and a low-price condition (cell phones). Participants were randomly assigned to one of the two experimental conditions (high vs. low price).

First, we provided a booklet called “consumer choice-making questionnaire” to the participants. In the beginning, it stated “In this questionnaire, I am interested in your choice between hedonic and utilitarian products and your thinking processing of making the choice. In the following pages, you will read about the attributes of a chosen product. Please read the information carefully and answer the questions that follow.”

Participants then read information about the two cell phones and two laptop computers. Each cell phone (laptop computer) was described as a combination of three hedonic attributes and three utilitarian attributes using the HED/UTI scale developed by Voss et al. (2003). The combination can be classified as mainly a hedonic or utilitarian product based on the study by Dhar and Wetenbroch (2000) and Okada (2005) as described in previous literature review sections.

After the participants finished the choice tasks, they were asked to list all the thoughts that came to their minds during the choice tasks. No time limit was set for this task. Finally, they were asked to fill out a questionnaire on demographics: gender, age, income, education, and household size.

#### Stimuli Construction

We conducted a pretest of the cell phone attributes to construct more realistic stimuli. We prepared a comprehensive list of approximately 50 attributes from real product manuals of the most popular cell phones in the market (e.g., Nokia, Samsung, and Motorola). We identified the top three influential attributes that offer hedonic benefits and the three most influential attributes that offer utilitarian benefits to construct the stimuli for the experiment. The same 120 participants were asked to rate all 50 attributes based on their importance. We first selected the 15 most influential attributes, and then picked 3 hedonic attributes and 3 utilitarian attributes. We created two levels of products (hedonic and utilitarian) by manipulating each attribute. One of the cell phone alternatives was high in style and attractiveness (hedonic) with a medium functionality (utilitarian). The other alternative was high in functionality (utilitarian) with a medium level of style and attractiveness (hedonic).

A similar pretest of laptop computers was conducted. We created a comprehensive list of 25 attributes from real product manuals of the most popular laptop computers in the market (e.g., IBM, APPLE, and SONY). We first selected 10 topmost influential attributes on purchase decisions, and then picked 3 hedonic attributes and 3 utilitarian attributes. We created two levels of products (hedonic and utilitarian) by manipulating each attribute. One of the laptop computer alternatives was high in style and attractiveness (hedonic) with a medium functionality (utilitarian). The other alternative was high in functionality (utilitarian) with a medium style and attractiveness (hedonic).

#### Stimuli Description

The hedonic cellphone attributes included oyster flip, changing phone colors, and programming new ring tones (yes/no). The utilitarian cellphone attributes included the level of network coverage (95% vs. 98%), battery capacity (2 days vs. 3 days), and sound clarity (medium vs. high).

The hedonic laptop attributes included screen size (regular vs. widescreen), changing laptop color, and flipping the screen. The utilitarian laptop attributes included the level of processing speed (1.5 GHz vs. 3 GHz), memory size (2 GB vs. 4 GB), and battery life (4 h vs. 6 h).

#### Results

**FIGURE 2:**
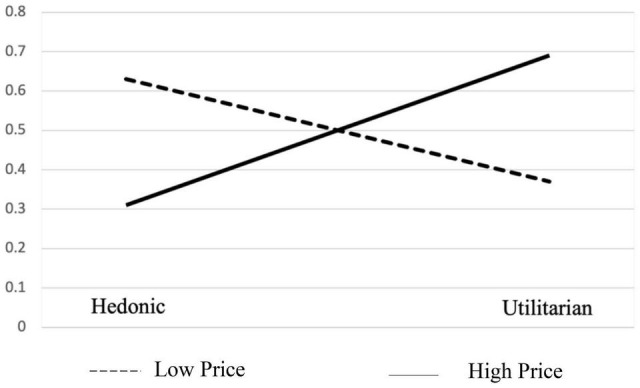
Choices at different price conditions.

To examine the effect of the product price on the consumer choices between hedonic and utilitarian alternatives, we performed a chi-square test. We predicted a preference for the hedonic item under the low-price condition and a preference for the utilitarian item under the high-price condition. And at the separate conditions, the difference between the preference for the hedonic item at a low price and the preference for the utilitarian item at a high price was significant. The results are consistent with our predictions in both conditions (Figure 2). When choosing a cell phone, 63% of the respondents selected the hedonic option. When choosing a laptop, 31% of respondents selected the hedonic option. There is a stronger preference for the hedonic item at the low-price condition, while there is a stronger preference for the utilitarian item at the high-price condition. The chi-square statistic was 12.06 and the *p*-value was 0.0005, all indicating a significant difference at the two price conditions. The results of this task are consistent with our predictions in H1 and H2. In low-price conditions, the loss risk is low; people are more likely to fulfill the promotion goals because they care more about gaining pleasure. While in high-price conditions, the loss risk is high; people are more afraid of losing than gaining, so they prefer to fulfill the prevention goal to be safe. Also, as we can see, the magnitude of difference for choices between hedonic and utilitarian products is bigger in the high-price condition than in the low-price condition. This is probably because of the loss aversion theory that loss looms larger than gain (Kahneman and Tversky, 1979). Harinck et al. (2007) found that “loss aversion does not exist in small payoff magnitudes,” which also explains our results. The results support Hypotheses 1 and 2.

### Suppressing Promotion and Prevention Goals in High- and Low-Price Conditions for Choices Between Hedonic and Utilitarian Products

#### Design and Task

The purpose of this study was to manipulate the regulatory focuses of the participants and measure the discounting effect of price on the consumer choices between hedonic and utilitarian products. A 2 (product benefits: hedonic vs. utilitarian) by 2 (price: high vs. low) by 2 (priming condition: promotion vs. prevention) between-subjects design was used. The same 120 undergraduate students participated for extra credit.

Following the procedure used in prior studies (see, e.g., Liberman et al., 1999; Idson et al., 2000), we randomly assigned participants in the two conditions. Participants in the promotion priming condition were asked to describe their current hopes and aspirations and how these differed from their hopes and aspirations as they grow up. Participants in the prevention priming condition were asked to describe their current duties and obligation and how these differed from their duties and obligation as they grow up. A blank page was provided, and participants were encouraged to spend about 10 min on the task.

Then, we assigned participants in two goal orientation conditions to both high-price choice tasks and low-price choice tasks. Participants were given the same choice tasks as used in the first study. The design and the instructions were the same as in Study 1. We followed the same steps and processes to construct the exact same cell phones and laptop computers for Study 2.

#### Results

**FIGURE 3:**
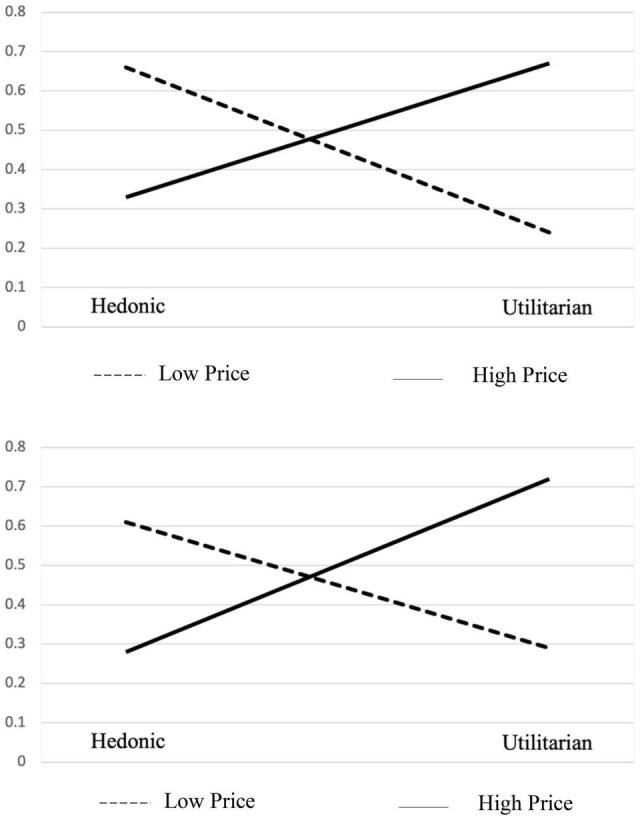
Choices at promotion/prevention priming conditions.

A chi-square test was performed to test the effect of price on the consumer choices between hedonic and utilitarian products under different regulatory focus conditions. We predicted that the preference of hedonic items in the low-price condition and the preference of utilitarian items in the high price condition will be attenuated by manipulating regulatory focus. The data showed a consistent effect of price as the results of Study 1 just with a smaller magnitude in both conditions. Thus, at the promotion priming condition, the preference for the hedonic item is higher than the results of Study 1 for both high- and low-price choice tasks. When choosing a cell phone, 66% of the respondents selected the hedonic option. When choosing a laptop, 33% of respondents selected the hedonic option. The chi-square statistic was 13.33 and the *p*-value was 0.0003, all indicating a significant difference at the two price conditions. At the prevention priming condition, the preference for the utilitarian item is higher than the results of Study 1 for both high- and low-price choice tasks. When choosing a cell phone, 61% of the respondents selected the hedonic option. When choosing a laptop, 28% of respondents selected the hedonic option. A chi-square test was conducted. The chi-square statistic was 13.47 and the *p*-value was 0.0002, all indicating a significant difference at the two price conditions (Figure 3). Significant differences in the two goal orientation conditions with two price level products indicate a significant main effect of price and interaction effect of price and goal orientation. We compared the choice preference between two goal orientations using the chi-square test as well. The resulting *p*-value was not statistically significant, indicating that the main effect of goal orientation is not significant.

### Suppressing Promotion and Prevention Goals by Mood in High- and Low-Price Conditions for Choices Between Hedonic and Utilitarian Products

#### Design and Task

The purpose of this study was to manipulate the regulatory focuses of the participants and measure the effect of mood on the consumer choices between hedonic and utilitarian products. We predicted that the effect of price on choice would be moderated by mood through regulatory focus. A 2 (product benefits: hedonic vs. utilitarian) by 2 (price: high vs. low) by 2 (mood condition: good vs. bad) between-subjects design was used. The same 120 undergraduate students participated for extra credit.

#### Mood Manipulation

A “life events survey” served as the mood induction. This exact mood induction has seen application in numerous behavioral studies and is effective in eliciting the desired moods in subjects (Strack et al., 1985). Participants were told that their descriptions of past “life events” would provide the basis for the development of a life-event survey. Subjects were asked to recall either a negative event (e.g., one that created strong unpleasant feelings) or a positive event (i.e., one that created strong positive feelings). The vividness of this experience was enhanced with several additional instructions by asking subjects to (a) visualize themselves in that situation, (b) try to experience all the feelings they had at the time, and (c) write down all the individual feelings they experienced (McFarland et al., 2007). Following this, subjects responded to positive (happy, pleasant, and cheerful) or negative (sad, blue, and gloomy) mood items, with additional filler items (reckless, focused, confused, bored, and interested) serving to mask the focus of the study on moods.

Then, we assigned participants in two mood conditions to both the high-price choice task and the low-price choice task. Participants were given the same choice tasks as used in the first study. The design and the instructions were the same as in Study 1. We followed the same steps and processes to construct the exact same cell phones and laptop computers for Study 2.

#### Results

**FIGURE 4:**
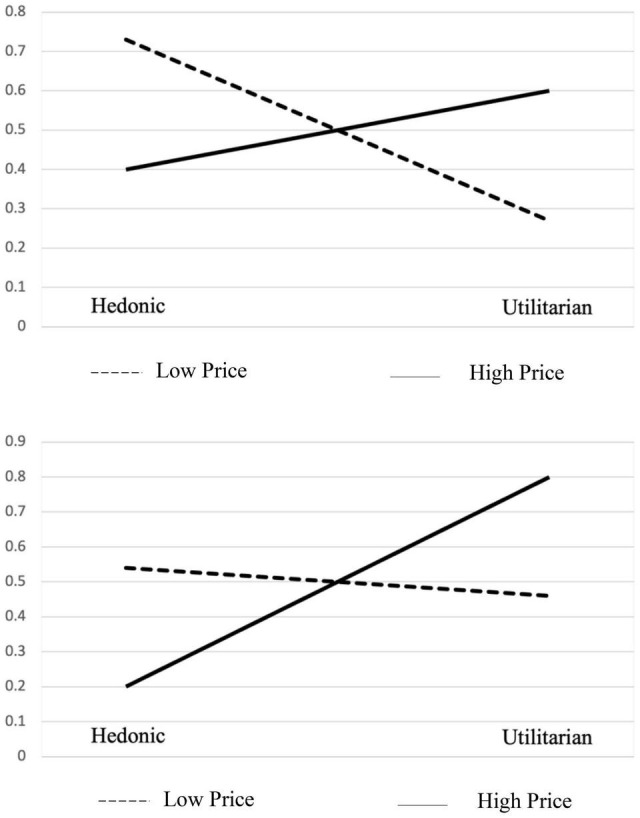
Choices in bad/good mood conditions.

A chi-square test was performed to examine the effects of price on the consumer choices between hedonic and utilitarian products under different mood conditions. We predicted that the preference of hedonic items in the low-price condition and the preference of utilitarian items in the high price condition will be attenuated by manipulating mood. The data showed a consistent effect of price as the results of study 1 just with a smaller magnitude in both conditions. Thus, in the bad mood condition, the preference for the hedonic item is higher than the results of Study 1 for both high- and low-price choice tasks. When choosing a cell phone, 73% of the respondents selected the hedonic option. When choosing a laptop, 40% of respondents selected the hedonic option. The chi-square statistic was 13.57 and the *p*-value was 0.0002, all indicating a significant difference at the two price conditions. At the good mood condition, the preference for the utilitarian item was higher than the results of Study 1 for both high- and low-price choice tasks. When choosing a cell phone, 46% of the respondents selected the utilitarian option. When choosing a laptop, 80% of respondents selected the utilitarian option. The chi-square statistic was 14.35 and the *p*-value was 0.0001, all indicating a significant difference at the two price conditions. Significant differences in two mood conditions with two price level products indicate a significant main effect of price and interaction effect of price and mood (Figure 4). We compared the choice preference between two mood conditions using the chi-square test also. The resulting *p*-value was statistically significant, indicating that the main effect of mood is also significant. The results support Hypothesis 4.

## Discussion

We demonstrated the different consumer preferences of the hedonic and utilitarian products at high/low-price conditions, and this can be attenuated by mood. The summary of the results is shown in Table 1. Overall, consumer purchase preference is affected by the promotion/prevention goal through regulatory focus. The promotion focus involves a state of eagerness to obtain advancement and gains, while the prevention focus involves a state of vigilance to assure safety and non-losses. These differences can be related to consumer perceptions of products over the hedonic and utilitarian dimensions.

**TABLE 1:** Summary of hypothesis testing results.

Hypothetic relationship	Expectations	Results	
**Experiment study**			
H1: Consumer prefers hedonic products at low price.	Positive	Positive	Supported
H2: Consumer prefers utilitarian products at high price.	Positive	Positive	Supported
H3: Prevention/promotion goals attenuate the effect.	Positive	Positive	Supported
H4: Mood conditions attenuate the effect.	Positive	Positive	Supported

### Theoretical Contribution

Building on the previous research (Kahneman and Tversky, 1979; Tversky and Kahneman, 1991; Dhar and Wetenbroch, 2000; Aaker and Lee, 2001; Chernev, 2004), we connected the gap between consumer purchase choices at different price conditions and with hedonic/utilitarian benefits through regulatory focus and loss aversion theory. Hedonic products are viewed as mostly experiential consumption with pleasure orientation for fun, whereas utilitarian products are viewed mostly as functional and instrumental consumption with practical orientation. The promotion focus is concerned with aspiration, advancement, growth, and accomplishment, whereas the prevention focus is concerned with security, responsibility, and safety. Thus, we connected different regulatory focuses with different product consumption: promotion focus offers a better fit with hedonic attributes, whereas prevention focus is likely to be more compatible with the more practical and conservative utilitarian attributes. We also investigated further the main and interacting effect of mood on consumer choices at different price conditions. The results from all studies showed that the regulatory focus of consumers dominates the underlying processing of choice-making theoretically and provides a possible solution to manipulate the consumer choice behaviors between hedonic and utilitarian goods by controlling their regulatory goals through price or mood factors.

### Managerial Implications

The findings of our study could have significant implications for marketers in various cases such as product design, especially in the retailing industry. Product designers and marketing managers are often compelled to make selections among a variety of attributes due to the budget constrain and time limitation of productions. If there is no budget constrain, the best solution should be to maximize all the product benefits. However, manufacturers can only invest the money in the highest return attributes. Often, product designers should emphasize more on one attribute than another or even select one over another. In this situation, our study should be able to provide some insights about the product designs for the managers.

For the retail stores, the findings of this study can also help them develop more effective promotions and store environments. Advertising has been a hot topic in consumer behavior research, and it has significant implications in the real market. We took a unique perspective of the role of mood in advertising effectiveness, from the regulatory focus aspect. When running the television spot for different products, marketers should consider selecting a cheer-up sadness ad for hedonic products and a maintaining happiness ad for utilitarian products. As for the store environment design, they can try to generate a more promotion goal-oriented environment for the hedonic products while generating a more prevention goal-oriented environment for the utilitarian products.

### Future Research Directions

First, the questionnaires in this study were answered by undergraduate students from a Midwest university, which may have its own limitations of a non-representative sample. Future research can use a more general population, which can represent the true market composition. Second, we used the same group of participants in all the three studies. In the future, different groups of participants can be used for external validity. Third, mediation analysis can be applied to test the direct and indirect effects of price on product choices and explore the underlying mechanism. Fourth, there are also so many other factors that might affect the consumer choices between hedonic and utilitarian products, such as gender, income, age, personal values, and innovativeness. Some might affect the choice through the regulatory focus while some others through different underlying thinking processes. We can either control these factors or explore their impact through further studies. Finally, we included laptops and cellphones as the product to be examined since the subject are familiar with them. As the participating subjects become different and being more representative, different products suiting the new conditions can be used. Also, different sets of products can be included for comparisons.

## Data Availability Statement

The original contributions presented in the study are included in the article/supplementary material, further inquiries can be directed to the corresponding author.

## Ethics Statement

Ethical review and approval were not required for the study on human participants in accordance with the local legislation and institutional requirements. Written informed consent from the participants was not required to participate in this study in accordance with the national legislation and the institutional requirements.

## Author Contributions

SZ and PC contributed equally to this work, they contributed to the empirical work, to the analysis of the results. YZ and FW contributed to overall quality, and supervision the part of literature organization and empirical work. FL contributed to developing research hypotheses and revised the overall manuscript. SZ was in charge of the article writing, communicating with the reviewers and made multiple revisions for final publication.

## Conflict of Interest

The authors declare that the research was conducted in the absence of any commercial or financial relationships that could be construed as a potential conflict of interest.

## Publisher’s Note

All claims expressed in this article are solely those of the authors and do not necessarily represent those of their affiliated organizations, or those of the publisher, the editors and the reviewers. Any product that may be evaluated in this article, or claim that may be made by its manufacturer, is not guaranteed or endorsed by the publisher.
